# Posterior tibial slope varies across functional tibial phenotypes but not CPAK categories: A radiographic analysis from the FP‐UCBM Knee Study Group

**DOI:** 10.1002/ksa.70135

**Published:** 2025-10-28

**Authors:** Edoardo Franceschetti, Giancarlo Giurazza, Alessandro Del Monaco, Marco Spatuzzi, Stefano Campi, Andrea Tanzilli, Pietro Gregori, Matteo Formica, Michele Paciotti, Rocco Papalia

**Affiliations:** ^1^ Fondazione Policlinico Universitario Campus Bio‐Medico Roma Italy; ^2^ Department of Medicine and Surgery, Research Unit of Orthopaedic and Trauma Surgery Università Campus Bio‐Medico di Roma Roma Italy; ^3^ Orthopedics Clinic IRCCS Ospedale Policlinico San Martino Genoa Italy; ^4^ Department of Surgical Science (DISC) University of Genoa Genoa Italy

**Keywords:** coronal plane alignment of the knee, functional tibial phenotypes, posterior tibial slope, sagittal knee alignment, total knee arthroplasty

## Abstract

**Purpose:**

The relationship between the coronal and sagittal planes of the knee remains largely unexplored. The aim of this study was to evaluate the correlation between Posterior Tibial Slope (PTS), Hirschmann's functional tibial phenotypes, CPAK categories, and their defining parameters, hypothesising that coronal and sagittal tibial parameters are interrelated.

**Methods:**

A retrospective analysis was conducted on preoperative radiographs from 204 patients (217 knees) undergoing primary TKA for end‐stage osteoarthritis between January 2023 and September 2023. PTS was measured on weight‐bearing true lateral radiographs with strict quality criteria. Coronal alignment variables—including arithmetic Hip‐Knee‐Ankle Angle (aHKA), Joint Line Obliquity (JLO) and Tibial Mechanical Angle (TMA)—were derived from full‐length standing radiographs, and CPAK categories and functional tibial phenotypes were assigned accordingly. Statistical analysis included Kruskal–Wallis tests and Spearman's correlations. Statistical significance was set at *p* < 0.05.

**Results:**

No significant differences in PTS were observed across CPAK categories (*p* = 0.501), aHKA categories (*p* = 0.103), or JLO categories (*p* = 0.906). In contrast, PTS differed significantly among functional tibial phenotypes (*p* = 0.004), with the highest values in VAR_TMA_6 (8.2° ± 3.4), VAL_TMA_6 (8.0° ± 2.7), and NEU_TMA_0 (7.7° ± 2.8), and lowest values in VAR_TMA_3 (5.7° ± 2.6) and VAL_TMA_3 (6.2° ± 2.1).

**Conclusion:**

PTS varies significantly across functional tibial phenotypes but not CPAK categories, suggesting that functional tibial phenotypes may better capture sagittal alignment variability. Incorporating PTS into phenotype‐based preoperative planning could help refine patient‐specific strategies in total knee arthroplasty.

**Level of Evidence:**

Level IV.

**Abbreviations**
aHKA: arithmetic hip‐knee‐ankle
CPAK: coronal plane alignment of the knee
FMA: femoral mechanical angle
ICC: intraclass correlation coefficients
JLO: joint line obliquity
LDFA: lateral distal femoral angle
MPTA: medial proximal tibial angle
PACS: picture archiving and communication system
PROMs: patient‐reported outcome measures
PTS: posterior tibial slope
SD: standard deviation
TKA: total knee arthroplasty
TMA: tibial mechanical angle

## INTRODUCTION

The knee joint has a complex three‐dimensional anatomy that requires a comprehensive understanding across all planes [10, 19, 20]. However, current research has predominantly focused on coronal plane alignment [11, 14, 21, 30], often neglecting the sagittal plane. Among sagittal parameters, the posterior tibial slope (PTS), which can vary up to 20° [5, 18, 31, 38], is a key determinant of knee biomechanics, influencing anteroposterior stability, physiological femoral rollback, and knee flexion [3, 7, 39, 40, 41]. Accurate reproduction of the slope is essential for proper tensioning of the PCL and to prevent excessive stress and wear on the posterior aspect of the polyethylene insert, while ensuring stable placement on dense subchondral bone [12, 15, 24, 36].

Recent studies have explored potential correlations between PTS and the Coronal Plane Alignment of the Knee (CPAK) classification [8, 17, 23, 33]. However, no significant associations have been identified. This may be due to the inherent limitations of the CPAK system [30], which is based on the combination of three categories of arithmetic Hip‐Knee‐Ankle (aHKA) angle and three categories of Joint Line Obliquity (JLO). It is therefore a qualitative rather than quantitative classification, which does not allow for differentiation between patients within the same category who may present markedly different values of medial proximal tibial angle (MPTA) and lateral distal femoral angle (LDFA) [10]. Since the posterior tibial slope is a purely tibial parameter, correlations with coronal alignment would be expected primarily with other tibial parameters. However, because CPAK merges tibial and femoral contributions within each category, such relationships may be obscured.

In contrast, the quantitative classification proposed by Hirschmann et al. [22]—including five femoral, five tibial, and five limb alignment phenotypes—offers a more detailed and anatomically grounded framework to overcome this limitation, but has never been explored in this context.

The aim of this study was to evaluate the correlation between posterior tibial slope and Hirschmann's functional tibial phenotypes, based on the hypothesis that coronal and sagittal plane parameters of the tibia are interrelated.

## MATERIALS AND METHODS

**Figure 1:**
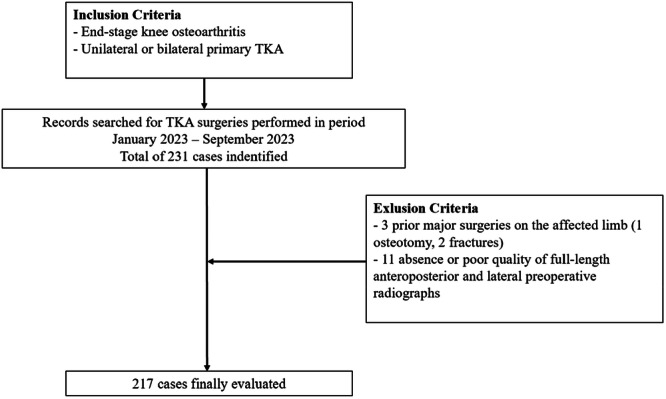
Flowchart depicting inclusion and exclusion criteria. TKA, total knee arthroplasty.

Institutional review board approval was obtained for this retrospective cohort study (IRB No. 32.19 OSS) which was reported in accordance with the Strengthening the Reporting of Observational Studies in Epidemiology (STROBE) guidelines and conducted in accordance with the Declaration of Helsinki. All patients provided informed consent. A retrospective analysis was conducted using prospectively collected preoperative radiographic data from patients undergoing primary Total Knee Arthroplasty (TKA) at our Institution (Fondazione Policlinico Universitario Campus Bio‐Medico) between January and September 2023. Inclusion criteria were: end‐stage knee osteoarthritis (Grade IV Kellgren–Lawrence), indication for unilateral or bilateral primary TKA. Exclusion criteria included a history of major surgery on the affected limb (e.g., osteotomies or fractures), absence or poor quality of full‐length anteroposterior and lateral preoperative radiographs (suboptimal tibial rotation or inadequate tibial shaft length). After applying inclusion and exclusion criteria, 204 patients (217 knees) were included in the final analysis (Table 1). A flowchart depicting patient inclusion and exclusion criteria is shown in Figure 1.

**Table 1:** Demographic characteristics.

Phenotype (*n* = 217)	Frequency (%)	Age ± SD (years)	BMI ± SD (kg/m^2^)	Gender (female, *n* [%])
1	47 (21.7)	71.3 ± 7.1	30.1 ± 4.2	22 (46.8)
2	82 (37.8)	71.1 ± 7.2	29.9 ± 5.1	52 (63.4)
3	19 (8.7)	71.9 ± 8.4	28.1 ± 4.1	14 (82.3)
4	30 (13.9)	71.3 ± 7.2	29.8 ± 4.3	14 (46.7)
5	14 (6.4)	69.4 ± 9.2	28.4 ± 5.2	7 (50.0)
6	19 (8.7)	70.2 ± 4.8	28.6 ± 3.2	11 (57.8)
7	3 (1.4)	67.8 ± 5.4	30.1 ± 4.9	2 (66.7)
8	1 (0.5)	74.0	30.5	1 (100.0)
9	2 (0.9)	68.0 ± 7.0	28.7 ± 8.1	2 (100.0)

Abbreviations: BMI, body mass index; SD, standard deviation.

### Radiographic analysis

For coronal plane measurements, full‐length, standing, weight‐bearing anteroposterior radiographs [13] were used to assess mechanical medial proximal tibial angle (MPTA), lateral distal femoral angle (LDFA), arithmetic HKA (aHKA), joint line obliquity (JLO) and tibial mechanical angle (TMA). Based on these values, CPAK and functional tibial phenotypes were assigned according to previously published methods [21, 25, 43, 44].

**Figure 2:**
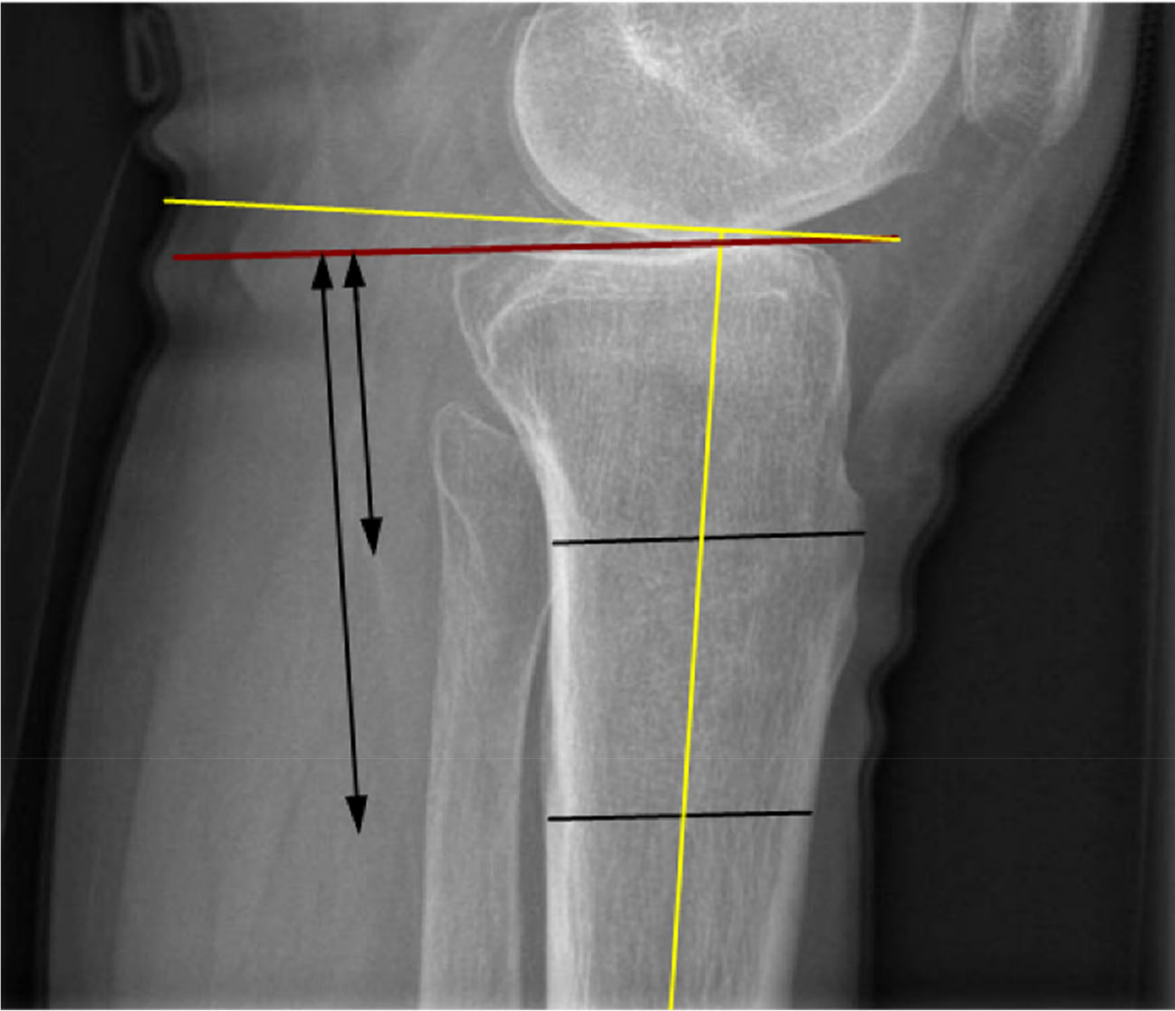
Posterior tibial slope. Black lines: anterior and posterior tibial cortices at 5 and 10 cm below the joint line; Yellow line: proximal anatomical axis of the tibia and its perpendicular; Red line: tangent connecting the anterior and posterior margins of the medial tibial plateau.

For sagittal plane evaluation, lateral short radiographs under weight‐bearing conditions were used to measure the posterior tibial slope. Strict exclusion criteria were applied to mitigate potential errors arising from suboptimal tibial rotation or variability in tibial shaft length captured in the radiographs. Only true lateral radiographs showing overlapping posterior femoral condyles, a centred knee joint, and at least 15 cm of the proximal tibia were deemed suitable for analysis [4, 12, 15]. PTS was measured according to the method described by Dejour et al. [9, 34] and was defined as the angle between the tibial proximal anatomical axis—obtained by connecting the midpoints of the anterior and posterior cortices at 5 and 10 cm below the joint line—and a line connecting the anterior and posterior limits of the medial tibial plateau (Figure 2).

All radiographic measurements were performed independently by two trained observers (MS, ADM) using Picture Archiving and Communication System (PACS)‐integrated tools, with results recorded to the nearest 0.5°. Intra‐ and inter‐observer reliability were assessed on a random sample of 50 patients, yielding intraclass correlation coefficients (ICC) of 0.94 and 0.89, respectively.

### Statistical analysis

Descriptive statistics were used to report mean values and standard deviations (SD) for all measured parameters. Normality of distributions was assessed using the Shapiro–Wilk test. The non‐parametric Kruskal–Wallis test was employed to evaluate differences in PTS across CPAK categories and functional tibial phenotypes. Spearman's correlation coefficients were calculated to investigate associations between PTS and coronal plane alignment variables. All analyses were performed using STATA 18 (StataCorp, College Station, TX), with significance set at *p* < 0.05.

## RESULTS

### Coronal plane parameters, CPAK classification and PTS

No significant correlations were found between PTS and MPTA, LDFA, aHKA, or JLO values (*p* > 0.05). No significant differences in PTS were observed when comparing varus, neutral, and valgus aHKA groups (*p* = 0.103), or different JLO groups (apex distal/neutral/proximal; *p* = 0.906). Similarly, no statistically significant differences were found in mean PTS across CPAK categories (*p* = 0.501) (Table 2).

**Table 2:** Relationship between PTS, CPAK phenotypes and their defining parameters (arithmetic hip‐knee‐ankle angle and joint line obliquity).

CPAK phenotype	Frequency (%)	Mean PTS (SD)	*p*‐value
1	47 (21.7)	7.1 (2.7)	0.501
2	82 (37.8)	6.5 (2.9)	
3	19 (8.7)	7.6 (2.2)	
4	30 (13.9)	6.6 (3.2)	
5	14 (6.4)	6.2 (2.0)	
6	19 (8.7)	7.7 (2‐8)	
7	3 (1.4)	5.9 (1.4)	
8	1 (0.5)	8.3 (0)	
9	2 (0.9)	6.1 (1.5)	
aHKA			
Varus	80 (36.9)	6.9 (2.9)	0.103
Neutral	97 (44.7)	6.5 (2.8)	
Valgus	40 (18.4)	7.5 (2.5)	
Joint line obliquity			
Apex distal	148 (68.2)	6.8 (2.8)	0.906
Apex neutral	63 (29.0)	6.9 (2.9)	
Apex proximal	6 (2.8)	6.4 (1.5)	

Abbreviations: aHKA, arithmetic hip‐knee‐ankle angle; CPAK, coronal plane alignment of the knee; PTS, posterior tibial slope; SD, standard deviation.

### Functional tibial phenotypes and PTS

**Figure 3:**
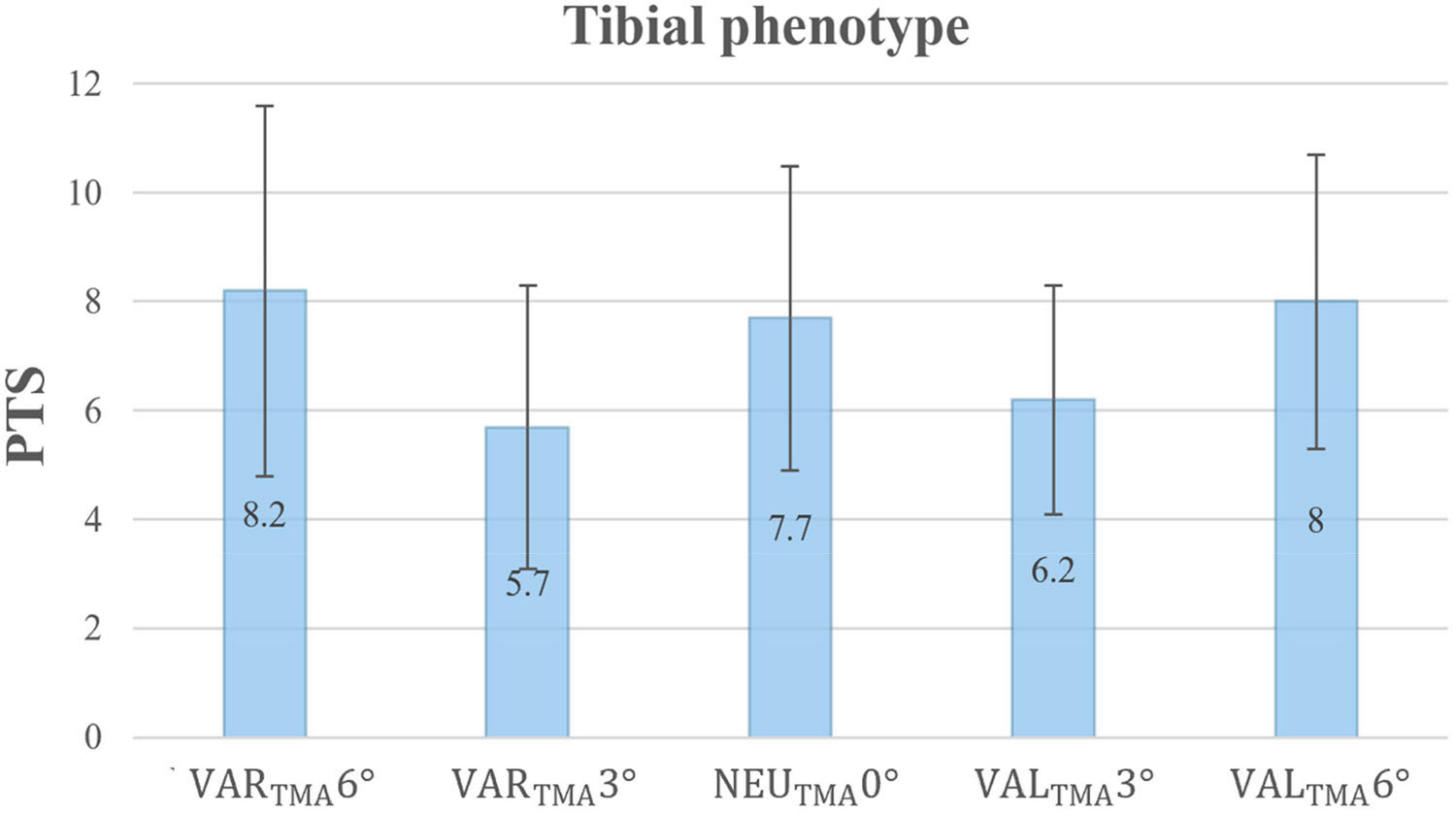
Posterior tibial slope (PTS) and functional tibial phenotypes. TMA, tibial mechanical angle.

PTS values demonstrated a non‐normal distribution across functional tibial phenotypes, with statistically significant differences between phenotypes. Specifically, the highest mean PTS values were observed in VAR_TMA_6 (8.24°, SD 3.40), VAL_TMA_6 (8.00°, SD 2.68), and NEU_TMA_0 (7.70°, SD 2.84) groups, whereas the lowest values were recorded in VAR_TMA_3 (5.72°, SD 2.55) and VAL_TMA_3 (6.23°, SD 2.11) (Table 3 and Figure 3).

**Table 3:** Relationship between PTS and tibial phenotypes.

TMA	Frequency (%)	Mean PTS (SD)	*p*‐value
VAR_TMA_6°	23 (10.6)	8.2 (3.4)	0.004
VAR_TMA_3°	40 (18.4)	5.7 (2.6)	
NEU_TMA_0°	70 (32.2)	7.7 (2.8)	
VAL_TMA_3°	49 (22.6)	6.2 (2.1)	
VAL_TMA_6°	35 (16.2)	8.0 (2.7)	

Abbreviations: PTS, posterior tibial slope; SD, standard deviation; TMA, tibial mechanical angle.

## DISCUSSION

The main finding of this study was that different Hirschmann's functional tibial phenotypes are associated with significantly different posterior tibial slope values. In particular, neutral tibias (NEU_TMA_0) and those presenting more pronounced varus (VAR_TMA_6) or valgus (VAL_TMA_6) alignment exhibited higher mean PTS compared to milder deformities (VAR_TMA_3 and VAL_TMA_3). In contrast, no significant associations were found between PTS and CPAK categories or their defining coronal parameters (MPTA, LDFA, aHKA and JLO), confirming previous findings in the literature [8, 17, 23]. Corbett et al. [8] in a computer tomography study on 509 Caucasian knees reported no correlation between CPAK categories and sagittal plane parameters. Similarly, Hiyama et al. [23] and Handa et al. [17] found little to no correlation between coronal and sagittal plane parameters in an Asian population, suggesting their inter‐independence. The absence of correlation between PTS and CPAK categories reinforces the notion that CPAK, although valuable in describing overall alignment, may lack the anatomical granularity necessary to guide decisions in the sagittal plane. Conversely, functional tibial phenotypes, by isolating tibial contributions to alignment, may offer a more suitable framework for assessing slope variability and its potential clinical implications. Our results showed that PTS is not uniformly distributed across the population but varies in relation to specific functional tibial phenotypes, supporting the idea that PTS may represent a biomechanical signature of each phenotype. To our knowledge, this is the first study in the literature to report this finding. Further research should investigate whether slope restoration targets should be phenotype‐dependent, whether integrating sagittal parameters into coronal phenotype classifications enhances preoperative planning and outcome prediction, and whether certain tibial phenotypes are more tolerant to slope adjustment than others.

Although the clinical influence of PTS variation remains debated, biomechanical studies have consistently demonstrated that deviations from native slope—either excessive reduction or increase—may negatively affect outcomes [1, 2, 29, 32]. Reducing PTS has been linked to limited postoperative flexion, increased PCL tension, and impaired rollback mechanisms [3, 7, 41, 42], while excessive PTS has been associated with elevated polyethylene stresses, implant wear, and increased risk of tibial component loosening [27, 28, 37]. Notably, although many studies have assessed the relationship between PTS and clinical outcomes after TKA, meaningful comparisons remain limited due to considerable heterogeneity in study designs, including inconsistent reporting of preoperative PTS, poor consideration of coronal alignment, differences in implant types (e.g., PCL‐retaining vs. PCL‐sacrificing), and diverse alignment philosophies [6, 27, 28, 37].

Moreover, the interplay between coronal and sagittal alignment must be considered in the broader context of flexion gap balance. Changes in posterior condylar offset, femoral component downsizing and PCL resection may typically require individualised slope adjustments to optimise soft tissue balance [26, 35, 39, 45].

This study is not without limitations. Its retrospective design and radiographic‐only analysis may not fully capture the three‐dimensional complexity of knee morphology, and the lack of functional outcome data limits clinical interpretation. Additionally, the exclusive inclusion of Caucasian patients from a single centre may restrict generalisability to other racial or ethnic populations [14, 23]. Future prospective studies incorporating postoperative alignment, implant data, ligament balance [16], and patient‐reported outcome measures (PROMs) are warranted to explore the clinical significance of phenotype‐specific PTS targets.

## CONCLUSION

PTS is not uniformly distributed across the population but varies in relation to specific functional tibial phenotypes, supporting the idea that PTS may represent a biomechanical signature of each phenotype. Functional tibial phenotypes influence posterior tibial slope, whereas CPAK categories do not. Integrating slope variability into phenotype‐based planning could help refine patient‐specific strategies in total knee arthroplasty.

## AUTHOR CONTRIBUTIONS

Edoardo Franceschetti and Stefano Campi were responsible for data collection and conceptualisation. Giancarlo Giurazza was responsible for writing of the manuscript and qualified as corresponding author. Alessandro Del Monaco and Marco Spatuzzi were responsible for the radiographic analysis. Andrea Tanzilli and Michele Paciotti were responsible for data analysis. Pietro Gregori and Matteo Formica were responsible for realisation of Figures and Tables. Rocco Papalia was responsible for reviewing and critically revise the manuscript. All authors have given final approval of the version to be published.

## CONFLICT OF INTEREST STATEMENT

The authors declare no conflicts of interest.

## ETHICS STATEMENT

The study was performed in accordance with the ethical standards as laid down in the 1964 Declaration of Helsinki and its later amendments. Institutional review board approval was obtained for this research (IRB n° 32.19 OSS). All patients provided legitimate informed consent.

## Data Availability

The data that support the findings of this study are available from the corresponding author, [G.G.], upon reasonable request.
